# A zebrafish screen for craniofacial mutants identifies *wdr68 *as a highly conserved gene required for endothelin-1 expression

**DOI:** 10.1186/1471-213X-6-28

**Published:** 2006-06-07

**Authors:** Robert M Nissen, Adam Amsterdam, Nancy Hopkins

**Affiliations:** 1 Center for Cancer Research and Department of Biology, Massachusetts Institute of Technology, 77 Massachusetts Avenue, Cambridge, MA 02139, USA; 2 Department of Biological Sciences, California State University Los Angeles, 5151 State University Drive, Los Angeles, CA 90032, USA

## Abstract

**Background:**

Craniofacial birth defects result from defects in cranial neural crest (NC) patterning and morphogenesis. The vertebrate craniofacial skeleton is derived from cranial NC cells and the patterning of these cells occurs within the pharyngeal arches. Substantial efforts have led to the identification of several genes required for craniofacial skeletal development such as the *endothelin-1 (edn1) *signaling pathway that is required for lower jaw formation. However, many essential genes required for craniofacial development remain to be identified.

**Results:**

Through screening a collection of insertional zebrafish mutants containing approximately 25% of the genes essential for embryonic development, we present the identification of 15 essential genes that are required for craniofacial development. We identified 3 genes required for hyomandibular development. We also identified zebrafish models for Campomelic Dysplasia and Ehlers-Danlos syndrome. To further demonstrate the utility of this method, we include a characterization of the *wdr68 *gene. We show that *wdr68 *acts upstream of the *edn1 *pathway and is also required for formation of the upper jaw equivalent, the palatoquadrate. We also present evidence that the level of *wdr68 *activity required for *edn1 *pathway function differs between the 1^st ^and 2^nd ^arches. Wdr68 interacts with two minibrain-related kinases, Dyrk1a and Dyrk1b, required for embryonic growth and myotube differentiation, respectively. We show that a GFP-Wdr68 fusion protein localizes to the nucleus with Dyrk1a in contrast to an engineered loss of function mutation Wdr68-T284F that no longer accumulated in the cell nucleus and failed to rescue *wdr68 *mutant animals. Wdr68 homologs appear to exist in all eukaryotic genomes. Notably, we found that the Drosophila *wdr68 *homolog *CG14614 *could substitute for the vertebrate *wdr68 *gene even though insects lack the NC cell lineage.

**Conclusion:**

This work represents a systematic identification of approximately 25% of the essential genes required for craniofacial development. The identification of zebrafish models for two human disease syndromes indicates that homologs to the other genes are likely to also be relevant for human craniofacial development. The initial characterization of *wdr68 *suggests an important role in craniofacial development for the highly conserved Wdr68-Dyrk1 protein complexes.

## Background

The widespread success of the vertebrate lineage owes partly to the acquisition of the neural crest (NC)-derived jaw apparatus of the craniofacial skeleton [1]. The adult craniofacial skeleton forms through replacing and elaborating the underlying NC-derived embryonic cartilaginous skeleton. The pathways and mechanisms that form the embryonic cartilaginous jaw are still poorly understood and many genes involved in this process remain to be identified. The rapid development of the jaw cartilages in the zebrafish and its amenability to forward genetics makes it an excellent model organism for studying the genetic basis of craniofacial development [2-4]; additionally, the use of retroviral insertional mutagenesis can facilitate the cloning of a large number of mutated genes [5,6].

Morphological development of the zebrafish pharyngeal cartilages has been described in detail [7-9]. Briefly, the chondrocytes that form these embryonic cartilages are derived from cranial NC cells that migrate from the neural tube to occupy transient embryonic structures called the pharyngeal arches. The NC cells in the 1^st ^arch give rise to the upper and lower jaw cartilages in all vertebrates examined. In zebrafish, the Meckel's cartilage (M) serves as the embryonic lower jaw and the palatoquadrate (PQ) serves as the embryonic upper jaw. The NC cells in the zebrafish 2^nd ^arch give rise to cartilages that support the jaw apparatus and anchor it to the neurocranium.

Extensive work in mice [10,11], chick [12], and zebrafish [13-15] has shed substantial light on the pathways that pattern the NC cells within an arch. The *endothelin-1 (edn1) *pathway is essential for patterning the NC cells that give rise to the lower jaw. Edn1 is a small peptide ligand secreted by the pharyngeal pouch ectoderm and endoderm cells as well as by the arch core mesoderm. As shown in other vertebrates, Edn1 signals through binding to a seven-transmembrane G protein-coupled receptor (EdnrA) expressed on the NC cells to regulate a downstream network of transcription factors [16-18]. In zebrafish, the downstream network includes members of the *distal-less *(*dlx*) gene family, *hand2 *and *bapx1 *[13,19,20]. Although recent evidence has shed light on how some of these downstream factors function in patterning the craniofacial skeleton, the upstream patterning events and pathways that lead to induction of *edn1 *are less clear. Notably, it has been reported that the transcription factor *tbx1 *functions upstream of *edn1 *signaling in the AB* zebrafish background [21]. Also, it has recently been reported that *tbx1 *is essential for Meckel's cartilage formation in *Xenopus *[22]. However, *tbx1*^-/- ^mice do not present Meckel's cartilage defects [23-25]. Importantly, *tbx1*^-/- ^mice do show reduction of the 1st arch-derived incus, a middle ear ossicle, indicating a role for *tbx1 *in 1st arch development [26]. Yet, *edn1*^-/- ^mice do not lack the incus [27]. Thus, the genes and mechanisms upstream of the *edn1 *pathway in Meckel's cartilage development remain unclear. Although it is possible that regulation of *edn1 *is different between mammals and either frog or fish, an alternative possibility is that a more universal and highly conserved pathway may await discovery.

To shed light on the pathways required for craniofacial patterning, we screened a collection of embryonic lethal insertional mutant zebrafish lines (for which the mutated gene had been identified in over 90% of cases) using the cartilage stain Alcian blue and inspected the mutants for defects in the craniofacial skeleton at 5 days post fertilization (dpf). We screened 306 genetic loci and identified 16 loci required for craniofacial development. We identified the mutated genes for 15 of these 16 loci. Two of the identified genes are known to be mutated in the human diseases Campomelic Dysplasia and Ehlers-Danlos syndrome and thus those two mutants might serve as zebrafish models for these diseases. Also among these mutants, we identified the *dirty south (dys) *mutant that harbors an insertion in the *wdr68 *gene. Wdr68 is a WD40 repeat domain protein of unknown function homologous to the petunia gene *AN11 *that regulates anthocyanin biosynthesis and it is also highly homologous to the Arabidopsis gene *TTG1 *that regulates root, shoot and leaf patterning events [28,29]. Wdr68 has been isolated from rabbit skeletal muscle in protein complexes containing two members of the Dual-specificity tyrosine-regulated kinase gene family, Dyrk1a and Dyrk1b [30]. *Dyrk1a*^-/- ^mice show general growth defects [31]. Notably, *DYRK1A *maps to a critical region for Down Syndrome (DS) in humans and over-expression of *Dyrk1a *in mice has been reported to cause DS-like neurological defects [32,33]. RNAi-mediated knockdown of *Dyrk1b *in the mouse myoblast cell line C2C12 causes a loss of myogenin expression and blocks in vitro differentiation of the cells into myotubes [34]. Thus, roles are emerging for *Dyrk1 *gene family members in various developmental processes [35,36].

Here, we show that *wdr68 *is required for lower jaw cartilage formation upstream of the *edn1*-signaling pathway. We also present evidence that *wdr68 *is required to form the upper jaw cartilage indicating that *wdr68 *activity is essential for all 1^st ^arch NC cell patterning. We demonstrate intracellular co-localization with nuclear Dyrk1a and present evidence suggesting that nuclear localization may be important for craniofacial patterning. Using an RNA rescue assay, we found that the fly *wdr68 *homolog CG14614 can rescue the *dys *mutant phenotype indicating conservation of *wdr68 *activity in animals lacking the NC cell lineage.

## Results

### Screening identified diverse genes involved in craniofacial development

**Figure 1:**
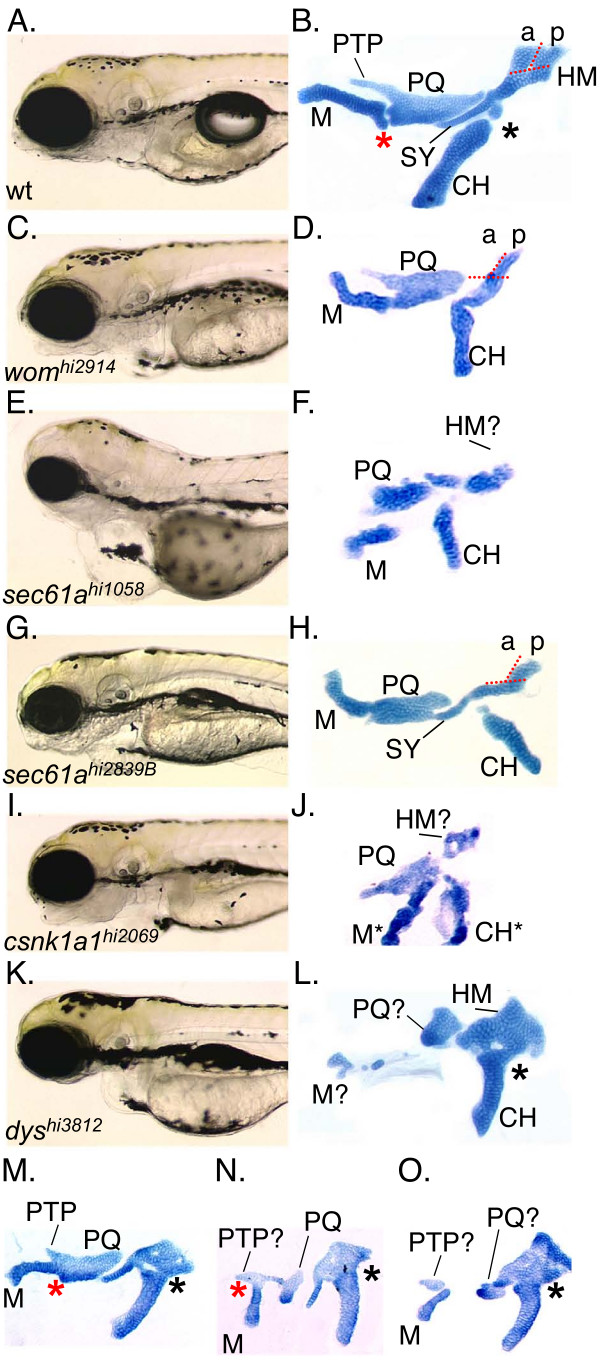
**The anterior arch mutants**. A, C, E, G, I, K), morphology at 4dpf. B, D, F, H, J, L), flat mounted alcian blue stained pharyngeal cartilages. Red asterisks indicate the location of the joint between the M and PQ cartilages. Black asterisks indicate the joint between the CH and hyosymplectic cartilages. A, B) wild type. C, D) HM reduction mutant *wom*^hi2914^. E, F) HM reduction allele *sec61a*^*hi1058*^. G, H) HM reduction allele *sec61a*^*hi2839B*^. I, J) anterior arch mutant *csnk1a1*^*hi2069*^. M* and CH* are readily identified but dysmorphic. K, L) Meckel's and PQ reductions in mutant *dys*^hi3812^. Most homozygous *dys/dys *animals show 'strong' reductions in the PQ such that only a posterior region of PQ is readily identifiable. M cartilage and the SY region are also strongly reduced. Joint fusion of the CH and HM region are also often present. M, N, O) Typically less than 5% of *dys/dys *animals show 'mild' reductions or joint fusions. M) Mild *dys/dys *animal showing only M-PQ and CH-HM joint fusions. N) Mild *dys/dys *animal showing intermediate PQ splitting in the presence of a well formed M that is fused to the anterior region of the PQ. O) Mild *dys/dys *animal showing complete PQ splitting but identifiable anterior and posterior PQ regions. The *nos*/foxI1 and *laz/pbx4 *mutants have been previously described and are therefore not shown.

A list of all the genes identified in the Hopkins lab by insertional mutagenesis screening has been published [6]. The insertional mutant collection can also be viewed online [37]. To identify genes required for craniofacial development we screened the insertional mutant collection at 5 days post fertilization using the cartilage stain Alcian blue. We inspected 306 recessive-lethal insertional mutant loci for defects in the craniofacial cartilages (Figure 1A, C). Briefly, the 1^st ^arch NC cells give rise to the zebrafish jaw that consists of a proximal palatoquadrate (PQ) that articulates with the distal Meckel's (M) cartilage. The 2^nd ^arch NC cells give rise to the hyosymplectic and ceratohyal (CH). The hyosymplectic is composed of a proximal hyomandibular (HM) region and a distal symplectic (SY) region. The hyosymplectic articulates with the distal CH and together these 2^nd ^arch-derived structures support the more anterior jaw by anchoring it to the neurocranium [8,9]. Through inspecting these cartilages, we identified 3 specific cartilage phenotype classes (Classes 1–3 listed in Table 1). To identify specific craniofacial development mutants, we excluded mutants that, in addition to anterior arch defects, also showed progressive loss of the posterior arches.

**Table 1:** Craniofacial development insertional mutants

Class 1- Anterior arch patterning		
Alleles	Gene	Phenotype
hi933, hi1126	*lazarus/pbx4 (lzr)*	arch fusions
hi1002, hi2069	*csnk1a1*	M, PQ defects
hi3812	*dirty south/wdr68 (dys)*	M, PQ defects
hi1058, hi2839B	*sec61a*	HM reduction
hi2914	*word of mouth/LOC321614-2 (wom)*	HM reduction
hi1321, hi1359A	*no soul/foxI1 (nos)*	HM reduction
hi3239, hi3747		
		
Class 2 – Chondrocyte differentiation		
Alleles	Gene	Phenotype

hi307, hi4205	*b3gat3*	reduced staining
hi954, hi3357	*uxs1*	reduced staining
hi1134	*jellyfish/sox9a (jef)*	reduced staining
hi1516, hi4063A	*b4galt7*	reduced staining
hi3378	*slc35d1*	reduced staining
hi3681	*atp6ap2*	reduced staining
hi4153	*srp68*	reduced staining
		
Class 3 – Cell morphology		
Alleles	Gene	Phenotype

hi1487	*gonzo/mbtps1 (goz)*	mildly poor stacking
hi1688, hi1934	*knypek/glp6 (kny)*	mildly poor stacking
hi1042	*old skool (odk)*	poor stacking

The anterior arch patterning class contains 6 mutant loci with specific patterning defects in the arch 1 derivatives, the arch 2 derivatives or both (Table 1, Figure 1). Studies on *lazarus*/*pbx4 *mutants revealed essential roles in neural tube development and maintaining separate neural crest streams [38]. Previous characterization of the *nos/foxI1 *mutant revealed the importance of cell survival signals in HM formation and additional screening identified two additional loci required for HM development, *word of mouth (wom) *and *sec61a *(Figure 1 C–H) [14]. We identified 2 alleles of *sec61a *and although the *sec61a*^hi1058 ^allele shows general defects that complicate analysis, the *sec61a*^hi2839B ^allele lacks the anterior portion of the HM (aHM) region but clearly retains the posterior HM (pHM) region (compare regions separated by red dotted lines in Figure 1A, H). Notably, the *wom *mutant also appears to lack the aHM region and retain the pHM region. In contrast, the *nos/foxI1 *mutant lacks both the aHM and pHM regions

The chondrocyte differentiation class contains 7 mutant loci with generally impaired overall chondrogenesis (Table 1). Among these mutants is the previously characterized *jellyfish/sox9a *(*jef) *mutant that is a zebrafish disease model for Campomelic Dysplasia [39,40]. We also identified a mutation in the glycosaminoglycan biosynthetic enzyme *b4galt7 *that represents a new zebrafish disease model for the progeroid form of Ehlers-Danlos Syndrome [see Additional file 1].

The cell morphology class contains 3 mutant loci that are required for proper chondrocyte morphology (Table 1). The *gonzo *and *knypek *mutants have been previously described [41,42]. We have not yet identified the gene disrupted in the *old skool (odk) *mutant [see Additional file 1].

We performed blast searches against the current human protein database using the predicted protein sequences for the 15 identified zebrafish genes. In 15/15 cases, a human homolog to the zebrafish protein was readily identified. The average amino acid sequence identity between the fish and human homologs was 77% and ranged from 47% to 99% identity (Table 2). Database analysis also revealed diverse cellular functions and protein domain architectures for the identified genes. Notably, 4 of the 7 chondrocyte differentiation mutants function in the glycosaminoglycan biosynthesis pathway highlighting the importance of this pathway in chondrogenesis and related disease states. Also, 2 of the 15 genes function in the ER protein secretory pathway, 2 genes function in Wnt signaling, 3 encode transcription factors and 2 encode WD40-repeat domain proteins. Thus, the genes we identified as essential for craniofacial development in the zebrafish appear to function in diverse cellular processes and are highly conserved in humans suggesting they may also be essential for human craniofacial development.

**Table 2:** Conservation of identified proteins between fish and human.

Zebrafish	Human	%Identity	Cellular process or protein domains
Pbx4	PBX4	81%	transcription factor
Csnk1a1	CSNK1A1	99%	S/T kinase in Hedgehog/Wnt signaling
Wdr68	WDR68	97%	WD40-repeats
Sec61a	SEC61A1	96%	ER protein export pathway
LOC321614-2	UNQ9342	93%	WD40-repeats
FoxI1	FOXI1A	47%	transcription factor
B3gat3	B3GAT3	58%	Glycosaminoglycan biosynthesis
Uxs1	UXS1	88%	Glycosaminoglycan biosynthesis
Sox9a	SOX9	70%	transcription factor
B4galt7	B4GALT7	65%	Glycosaminoglycan biosynthesis
Slc35d1	SLC35D1	80%	Glycosaminoglycan biosynthesis
Atp6ap2	ATP6AP2	68%	integral membrane protein
Srp68	SRP68	79%	ER protein export pathway
Mbtps1	MBTPS1	85%	sterol/lipid metabolism
Glp6	GPC6	55%	Wnt signaling

### Phenotype of the *dirty south (dys) *mutant and identification of *wdr68 *as the mutated gene

The *dirty south (dys) *mutants display variably reduced M and PQ cartilages with variable reductions in the CH as well (Figure 1K, L). The mutant was named in reference to the variable lower jaw cartilage defects. Through inspecting large numbers of Alcian blue stained *dys *mutant animals we found that most *dys *mutants displayed the 'strong' phenotype of severe reductions in both the M and PQ with small clusters of cartilage cells possibly derived from either the M or PQ or both often present (M? and PQ in Figure 1L). Although in most *dys *mutant animals the CH was present, we occasionally observed *dys *mutant animals that lacked one of the two bilaterally symmetric CH cartilages (data not shown). In fact, we often observed asymmetric defects where cartilages on one side would appear more severely reduced than on the contra-lateral side (data not shown). Also, less than 5% of observed *dys *mutants showed mild defects instead of the more severe losses described above. These 'mild' defect animals tended to show only simple joint fusions between otherwise relatively normal M and PQ as well as CH and HM cartilages (Figure 1M, red asterisk indicates 1^st ^arch joint region, black asterisk indicates 2^nd ^arch joint region). The PQ is composed of two distinct regions. The ptergoid process (PTP) is a thin rod of chondrocytes that extends anteriorly from the planar body of the PQ. A small percentage of *dys *mutants showed restrictions of the planar mid-region of the PQ with what appears to be an identifiable anterior PTP region and posterior planar PQ body region (Figure 1N). The distal M cartilage was always observed in these animals, albeit fused to the anterior region of what might be the PTP. Occasionally, we observed what appears to be complete splitting of the PQ planar body (Figure 1O).

**Figure 2:**
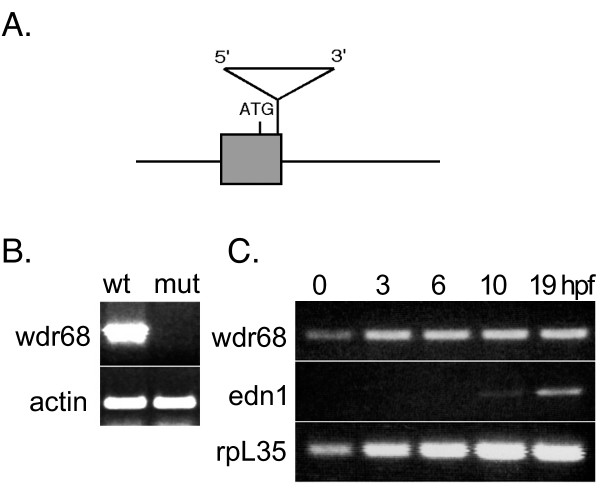
**The *dys*^hi3812 ^mutant is a null**. A) Diagram indicating the location of the *dys*^hi3812 ^proviral insert in the first exon of the *wdr68 *gene. B) RT-PCR analysis of wild type siblings and *dys/dys *animals showing that wild type *wdr68 *transcripts are not detectable in the *dys *mutants indicating that the mutant is a null allele. C) RT-PCR analysis reveals a maternal supply of *wdr68 *transcripts. cDNA from wild type embryos at zero = unfertilized, 3 hpf, 6 hpf, 10 hpf, and 19 hpf. As negative control, *edn1 *transcripts are only detected after 10 hpf. Transcripts for the ribosomal gene *rpL35 *are present at all stages examined.

The *dys *locus encodes the 342 amino acid protein Wdr68 [6]. Genome database searches revealed only one *wdr68 *gene in the human, mouse, and fish although we cannot rule out the possibility that the genome databases are incomplete. The insertion is in the first exon of the *wdr68 *gene, 131 nucleotides downstream from the predicted translation initation site (Figure 2A). RT-PCR analysis revealed that *dys*^hi3812 ^is a null mutant (Figure 2B). Notably, *wdr68 *transcripts are readily detected by RT-PCR in wild type animals at all developmental stages including unfertilized oocytes (Figure 2C). As a negative control, *edn1 *transcripts, which are not detectable by ISH until late somitogenesis stages, could not be detected by RT-PCR in unfertilized oocytes or in samples earlier than 10 hpf (Figure 2C). Although the observed cartilage phenotype variation outlined in figures 1 L–O might be due to residual maternal *wdr68 *activity in the *dys *mutants, we cannot rule out the possibility that this observed phenotypic variation may result from the independent segregation of an unknown number of modifier loci.

### The *wdr68 *gene is expressed ubiquitously during early development

**Figure 3:**
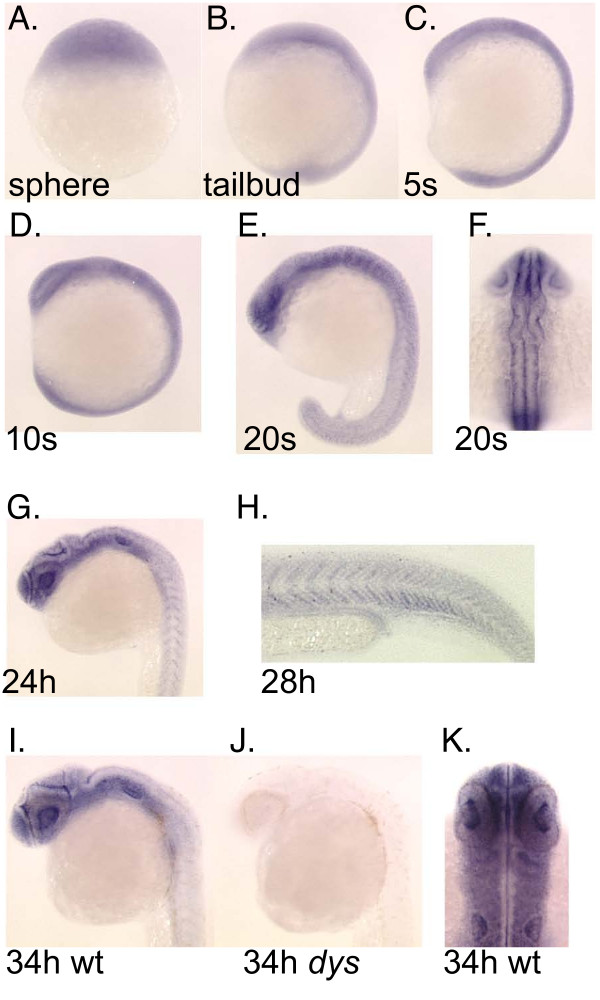
**Expression pattern for the *wdr68 *gene during early development**. A) Transcripts are present in all cells of the animal at sphere stage. B) Ubiquitous expression at tailbud stage. C) Ubiquitous expression at 5 somites stage. D) Ubiquitous expression at 10 somites stage. E) Ubiquitous expression with modest enrichment in the forebrain and hindbrain at 20 somites stage. Also note emerging patterned expression in the developing somites. F) Dorsal view of same 20 somites stage animal indicating ubiquitous expression. G) Ubiquitous expression with moderate enrichment in the head region at 24 hpf. H) Higher magnification image of a 28 hpf animal to show patterned expression in somites. I, J) Wild type sibling and *dys/dys *homozygous mutant at 34 hpf. I) Enriched expression in the head region with much lower expression in somites in a 34 hpf wild type sibling animal. J) Absence of detectable *wdr68 *transcripts in the *dys/dys *homozygous mutant sibling. K) Dorsal view of head region of 34 hpf animal showing near ubiquitous expression in developing head structures.

Consistent with the RT-PCR data indicating the presence of *wdr68 *transcripts at all early stages of development, we found by whole-mount in situ hybridization (ISH) ubiquitous expression of *wdr68 *at sphere, tailbud, 5 somites and 10 somites stages (Figure 3A, B, C, D). By the 20 somites stage, some enrichment of transcripts in the forebrain and hindbrain regions could be detected and patterned expression in the developing somites was also apparent (Figure 3E). Also, transcripts could be observed lateral to the neural tube in a region that would correspond to the post-migratory NC cells at this stage (Figure 3F). By 24 hpf, *wdr68 *transcripts were significantly enriched in the head region relative to the tail region (Figure 3G). By 28 hpf, restricted expression of *wdr68 *was evident in the developing somites (Figure 3H). By 34 hpf, *wdr68 *transcripts were expressed strongly in almost all head structures and only expressed at very low levels in the tail region (Figure 3I, K). ISH analysis on *dys *mutant animals was not able to detect any *wdr68 *expression consistent with the RT-PCR results indicating that the *dys*^hi3812 ^allele encodes a null mutation of the *wdr68 *gene (Figure 3J). Notably, *wdr68 *transcripts were detected from sphere stage through to 34 hpf in all regions of the embryo currently known to play roles in craniofacial development including the pharyngeal pouches.

**Figure 4:**
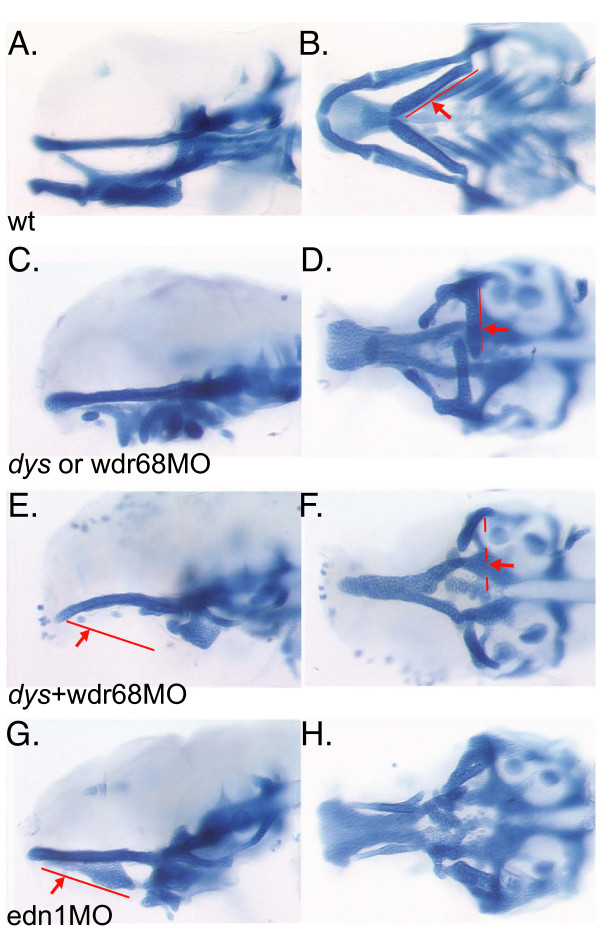
***wdr68 *activity is required for all 1^st ^arch cartilage formation**. A), C), E), G) Lateral views of Alcian blue stained animals at 4dpf. B), D), F), H) Ventral views of Alcian blue stained animals at 4dpf. In panels D, F, and H the posterior arches were removed to enhance clarity for observing the anterior arch defects. A), B) Wild type sibling. C), D) Representative 'strong' phenotype *dys/dys *animal similar to that shown flatmounted in figure 1 panel D with incomplete loss of M and PQ cartilages. The *wdr68*MO when injected into wild type animals yields a phenotype very similar to that shown here in panels C and D. E), F) Injecting *dys/dys *animals with antisense *wdr68*MO causes complete loss of the PQ, M and CH but does not substantially reduce the HM region. G), H) Representative *edn1*MO knockdown animal lacking the M and CH but retaining the PQ and HM regions.

To further confirm that the *dys *mutant phenotype is caused by the proviral insertion disrupting the *wdr68 *locus, we designed two translation blocking antisense morpholino oligonucleotides (*wdr68*MO and *wdr68*MO2) to the *wdr68 *transcript. We found that both gave similar results and that both gave the similar range of variable M, PQ, SY and CH defects as were observed for the *dys *mutant (wildtype controls in Figure 4A, B and mutants or morphants in Figure 4C, D and data not shown). The images in Figure 4 depict the craniofacial apparatus in whole-mount embryos in contrast to the dissected flat-mounted cartilages shown in Figure 1.

### *wdr68 *activity is required for all 1^st ^arch cartilage formation

In performing titrations of the *wdr68*MO we noted that low concentrations tended to cause only mild cartilage joint fusion defects while higher concentrations were more similar to the defects observed in *dys*^hi3812^*/dys*^hi3812 ^homozygotes and occasionally yielded more severe reductions in the PQ and CH regions. Since RT-PCR analysis indicated that *wdr68 *transcripts are maternally supplied we reasoned that zygotic *dys *mutants probably harbor some residual amount of maternally supplied *wdr68 *activity. Likewise, antisense morpholino knockdowns are not 100% efficient at blocking translation and therefore residual *wdr68 *activity would likely also be present in the knockdown animals. Thus, we injected clutches of embryos from *dys*^hi3812^/+ parental crosses with the *wdr68*MO (*dys+wdr68*MO) and stained the cartilages with Alcian blue in an attempt to reveal more clearly what might be the phenotype for a more complete loss of *wdr68 *activity. We found that approximately 3/4 of the embryos were similar to wild type animals injected with the *wdr68*MO and showed the range of phenotypes typically observed for *dys*/*dys *homozygotes leading us to suspect they represent the expected mendelian 3/4 of +/+ and *dys*/+ animals (Figure 4C, D and data not shown). In contrast, we found that approximately 1/4 of the injected animals nearly completely lacked the M, PQ, SY and CH cartilages (Figure 4E, F). A negative control morpholino had no effect in wild type embryos and did not significantly exacerbate the cartilage defects in clutches of embryos from *dys*^hi3812^/+ parental crosses (data not shown). We found similar results using *wdr68*MO2 (data not shown).

We compared the *dys+wdr68*MO animals to animals injected with a previously characterized *edn1 *translation blocking morpholino (*edn1*MO) that is reported to nearly fully phenocopy the defects present in *edn1 *mutant zebrafish [43]. Similar to previous reports we found that *edn1 *is required for the formation of the M, SY and CH cartilages but not for the PQ or HM regions (Figure 4G, H). In contrast, we found that *dys *mutants also present defects in the PQ that seem to affect the anterior PTP region more strongly than the posterior planar PQ region (Figure 1L). Attempts to reduce *wdr68 *activity from maternally supplied transcripts through generating *dys+wdr68*MO animals showed almost complete loss for the M, PQ, SY and CH (Figure 4E, F). Thus, *wdr68 *activity appears to be required for all *edn1*-dependent cartilages plus the PTP and PQ regions.

### *wdr68 *activity is not required for normal NC cell migration

**Figure 5:**
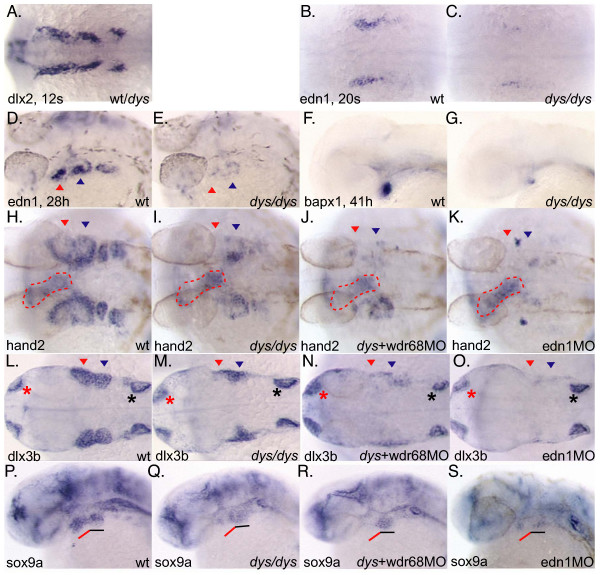
***wdr68 *acts upstream of the *edn1 *pathway**. A) In situ hybridization (ISH) with *dlx2 *on 12 somites stage embryos. Wild type and *dys/dys *mutant animals were indistinguishable. B) ISH on 20 somites stage wild type sibling animal with *edn1 *probe. C) *dys/dys *homozygotes display reduced edn1 expression at 20 somites stage. D) ISH on 28 hpf wild type sibling animal with *edn1 *probe. E) *dys/dys *homozygotes display reduced *edn1 *expression at 28 hpf. F) ISH on 41 hpf wild type sibling with *bapx1 *probe. G) *dys/dys *homozygotes display reduced *bapx1 *expression. H, I, J, K, L, M, N, O) Red arrowhead indicates 1^st ^arch expression domain, blue arrowhead indicates 2^nd ^arch expression domain. H) ISH on 28 hpf wild type sibling with *hand2 *probe. I) *dys/dys *homozygotes display reduced 1^st ^arch *hand2 *expression, red arrowhead. Red dotted outline indicates expression in developing heart. J) *dys+wdr68*MO animals display reduced *hand2 *expression in both 1^st ^and 2^nd ^arches, red and blue arrowheads, respectively. K) *edn1*-MO animals lack *hand2 *expression in both the 1^st ^and 2^nd ^arches. L, M, N, O) Red asterisk indicates olfactory expression domain. Black asterisk indicates ear expression domain. L) ISH on 28 hpf wild type sibling with *dlx3b *probe. M) *dys/dys *homozygotes display reduced 1^st ^arch *dlx3b *expression, red arrowhead. N) *dys+wdr68*MO animals display reduced *dlx3b *expression in both 1^st ^and 2^nd ^arches, red and blue arrowheads, respectively. O) *edn1*-MO animals lack *dlx3b *expression in both the 1^st ^and 2^nd ^arches. P, Q, R, S) Red underline indicates 1^st ^arch region for *sox9a *expression. Black underline indicates 2^nd ^arch region for *sox9a *expression. P) ISH with *sox9a *probe on 28 hpf wild type sibling. Q) Reduction of 1^st ^arch *sox9a *expression without significant effect on 2^nd ^arch *sox9a *expression in *dys/dys *animals. R) Injecting *dys *animals with *wdr68*MO caused further reduction of 1^st ^arch *sox9a *expression without substantial effects on 2^nd ^arch *sox9a *expression. S) *edn1*-MO animals show reduced but still detectable *sox9a *expression in both the 1^st ^and 2^nd ^arches.

Since defects in NC cell migration might lead to the loss or malformation of craniofacial elements, we used a *dlx2 *probe in whole-mount ISH to determine whether cranial NC cell migration occurs normally in *dys *mutant animals. We could not detect any alterations in the migration of the 3 cranial NC cell streams that fill the 1^st^, 2^nd ^and posterior arches at the 12 somites stage suggesting that neither cranial NC cell induction or migration is severely altered in *dys *mutants (Figure 5A).

### *wdr68 *activity is required for endothelin-1 expression

Because loss of *wdr68 *activity caused defects in all *edn1*-dependent cartilage elements plus the PQ, we examined whether *wdr68 *might act upstream of the *edn1 *pathway [43]. Using an *edn1 *probe in whole-mount ISH, we found that while *edn1 *was readily detected in wild type siblings at the 20 somites stage, *dys*^hi3812^*/dys*^hi3812 ^animals had severely reduced *edn1 *transcript levels (Figure 5B, C). To rule out the possibility that there might be a mild delay in *edn1 *expression, we also examined expression at 28 hpf and found expression to also be severely reduced at this later stage (Figure 5D, E). We left the 28 hpf animals unbleached to indicate the relatively normal development of the neural crest that give rise to melanocytes to further indicate the absence of a general developmental delay in *dys *mutant animals (Figure 5D, E). Notably, low and slightly variable levels of *edn1 *transcripts were detected in the *dys *mutants at both the 20 somites stage and later at the 28 hpf stage. Variation in the residual levels of *edn1 *transcripts may account for some of the observed phenotypic variation (Figure 1L–O). For additional evidence that *wdr68 *is required for *edn1 *pathway activity we also examined several downstream components of the *edn1 *pathway. *bapx1 *is a transcription factor required for induction of the jaw joint that is *edn1*-dependent [19]. In situ hybridization with *bapx1 *probe showed that while readily detected in wild type siblings, *dys*^hi3812^*/dys*^hi3812 ^animals had severely reduced *bapx1 *transcript levels (Figure 5F, G). The transcription factors *hand2 *and *dlx3b *are also *edn1*-dependent and in situ hybridization likewise showed that *dys*^hi3812^*/dys*^hi3812 ^animals had reduced transcript levels (Figure 5H, I and Figure 5L, M). Notably, only the arch-specific expression domains for both *hand2 *and *dlx3b *were affected indicating that a general delay in embryonic development cannot explain the absence of expression in the arches (Figure 5H, I, J, K the red dotted outline indicates the unaffected expression of *hand2 *in developing heart. Figure 5L, M, N, O the red and black asterisks indicate the unaffected olfactory and otic expression domains of *dlx3b*, respectively). Also, the 1^st ^arch domains for both markers was more reduced than the 2^nd ^arch domains perhaps explaining the relatively milder 2^nd ^arch derived CH cartilage reductions. (red arrowheads indicate 1^st ^arch, blue arrowheads indicate 2^nd ^arch, Figures 5H, I and 5L, M). Examining *edn1 *expression at this relatively late developmental stage, 28 hpf, revealed a similar difference with 1^st ^arch *edn1 *expression more reduced than 2^nd ^arch *edn1 *expression (red arrowheads indicate 1^st ^arch, blue arrowheads indicate 2^nd ^arch, Figure 5D, E).

Analyzing the cartilages of *dys+wdr68*MO animals suggested that *wdr68 *activity is required for all *edn1*-dependent cartilage development. To more precisely explore this possibility, we generated *dys+wdr68*MO animals and processed them for ISH analysis to determine whether the residual expression of *hand2 *and *dlx3b *we observed in the 2^nd ^arch is *wdr68*-dependent. We found that 2^nd ^arch expression of *hand2 *and *dlx3b *was consistently more reduced in approximately 1/4 of the *dys+wdr68*MO animals. The further reduction of 2^nd ^arch expression is not due to a non-specific general developmental delay because expression of *hand2 *in the developing heart was not affected and development of the NC cell-derived melanocytes was not affected in the animals analyzed for *hand2 *expression (dotted outlined structure is heart expression, Figure 5H–K). Likewise, expression of *dlx3b *in the developing olfactory and otic domains was not substantially affected in the *dys+wdr68*MO animals (red and black asterisks, respectively, Figure 5L–O). We also compared the expression of *hand2 *and *dlx3b *to *edn1*MO animals and found that *dys+wdr68*MO animals more closely resemble *edn1*MO animals than do the *dys *mutants but that directly blocking *edn1 *expression does yield more complete reductions suggesting that *dys+wdr68*MO animals still harbor some residual *edn1 *signaling (Figure 5K, O). We also found that *wdr68 *activity is similarly required for the arch-specific expression domains of *dlx4a, dlx4b, dlx5a *and *dlx6a *(data not shown). Expression of the *dlx *genes was also *edn1*-dependent (data not shown). Collectively, these data indicate that *wdr68 *activity is required for *edn1 *expression and its downstream targets.

In zebrafish, as in mammals, chondrocyte differentiation requires the activity of a *sox9 *transcription factor [39,40]. To determine whether *wdr68 *activity is also required for chondrocyte differentiation, we examined expression of the zebrafish *sox9a *gene. Like the other arch markers, we found that 1^st ^arch expression was more reduced than 2^nd ^arch expression in *dys *mutants (1^st ^arch red underline, 2^nd ^arch black underline, Figure 5P, Q, R). Consistent with *edn1*MO animals having a relatively normal PQ, we detected some residual *sox9a *expression in the 1^st ^arch of those animals (red underline, Figure 5S). In all cases, residual *sox9a *expression in the 2^nd ^arch region likely reflects the relatively normal formation of the HM region (Figure 1L).

The *tbx1 *gene has been shown to regulate *edn1 *expression in the AB* zebrafish line [21]. However, loss of *tbx1 *causes less severe defects in the Tubingen zebrafish line, perhaps due to differences in modifier loci between the AB* and Tubingen lines [44]. Since the insertional mutant lines were generated and are maintained on TAB5 and TAB14, two lines derived from a cross between Tubingen and AB* [45], we cannot readily predict the role for *tbx1 *in the lines used in this study. Regardless, we examined the expression of *tbx1 *in *dys *animals and found normal expression at 24 hpf and at most only modest reduction of *tbx1 *expression in the pharyngeal region at 30 hpf (data not shown). Therefore, *wdr68 *does not appear to be essential for *tbx1 *expression and *tbx1 *expression alone is not sufficient for *edn1 *expression in our lines.

### Arch identity underlies the differential sensitivity to *wdr68 *activity

**Figure 6:**
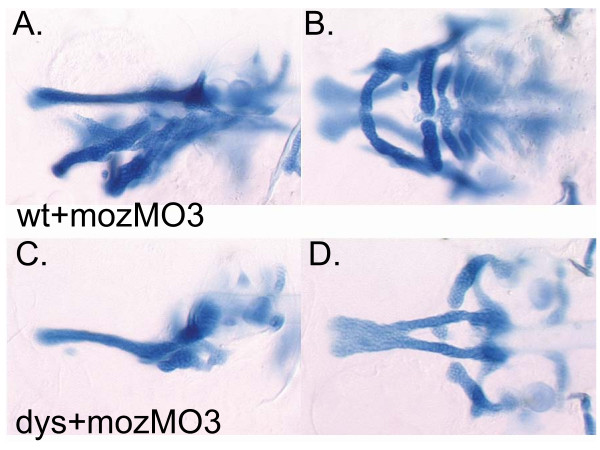
**Transformation of 2^nd ^arch identity transforms 2^nd ^arch *wdr68 *sensitivity**. A, C) Lateral views at 4dpf. B, D) Ventral views at 4dpf. A, B) Wild type animal injected with mozMO transforms 2^nd ^arch cartilage elements into mirror image duplicates of 1^st ^arch cartilages. C, D) Injecting *dys *animals with *moz*MO reveals similar cartilage losses as in maternal *wdr68 *knockdowns shown in (Fig. 4E, F) but with further reduction of HM region presumably through mirror image duplication of the missing 1^st ^arch cartilages.

The observation that the 1^st ^arch derived M and PQ cartilages were consistently more severely reduced than the 2^nd ^arch derived CH and HM cartilages in *dys*^hi3812^*/dys*^hi3812 ^animals suggested that perhaps 1^st ^arch patterning requires more *wdr68 *activity than 2^nd ^arch patterning. ISH analysis of *hand2 *and *dlx3b *expression also suggests that 1^st ^arch patterning requires more *wdr68 *activity than 2^nd ^arch patterning. Consistently, injecting animals with antisense translation blocking morpholinos increased the severity of 2^nd ^arch cartilage and gene expression phenotypes. To test whether *wdr68 *activity is differentially required between the 1^st ^and 2^nd ^arches, we hypothesized that if the 1^st ^arch structures require higher levels of *wdr68 *activity than the 2^nd ^arch structures, then transformation of the 2^nd ^arch into a duplication of the 1^st ^arch would result in near complete absence of all jaw cartilages. The NC cells within the 1^st ^arch do not express the *hox *transcription factor genes that are expressed by NC cells within the 2^nd ^arch. Loss of *hox *transcription factor activity in the 2^nd ^arch causes transformation into 1^st ^arch identity. Likewise, it has been shown that loss of the *hox *transcription factor co-activator *moz/myst3 *causes transformation of the 2^nd ^arch into a duplication of the 1^st ^arch [46-51]. Therefore, we injected clutches of embryos from *dys*^hi3812^/+ parental crosses with an antisense morpholino against *moz/myst3 (moz*MO3) and stained for cartilages. In approximately 3/4 of the animals, we observed transformation of the 2^nd ^arch derivatives into a duplication of the 1^st ^arch cartilages (Figure 6A, B). These animals were phenotypically indistinguishable from wild type TAB5 and TAB14 animals injected with *moz*MO3 consistent with the notion that these animals represent the +/+ and *dys*^hi3812^/+ siblings as expected based on mendelian genetics. The slightly less complete nature of these transformations relative to results previously reported might be due to differences between the zebrafish lines used in this study versus the previously reported study [51]. In contrast, approximately 1/4 of the animals lacked virtually any jaw cartilages (Figure 6C, D). We reason, based on mendelian ratios, that these animals are likely to be the *dys*^hi3812^/*dys*^hi3812 ^animals. The strong reduction of the transformed 2^nd ^arch derivatives is consistent with a differential requirement for *wdr68 *activity along the A/P axis. These results together with the ISH data on *edn1, hand2 *and *dlx3b *support a model in which the level of *wdr68 *activity required for 1^st ^arch-associated edn1 expression is higher than that required for 2^nd ^arch-associated edn1 expression explaining why *dys/dys *animals display relatively milder 2^nd ^arch defects than would be predicted for a gene required upstream of *edn1*.

### Wdr68 protein localizes to the cell nucleus

**Figure 7:**
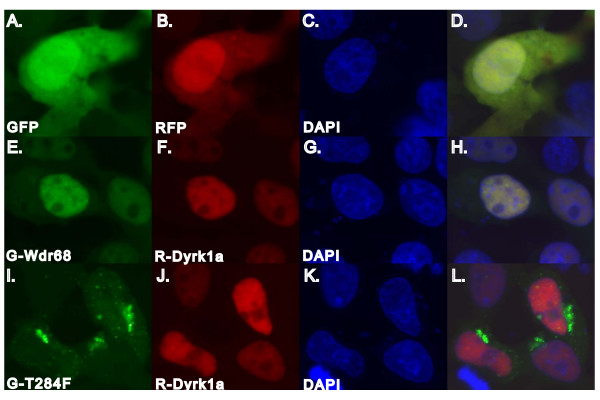
**The Wdr68 protein co-localizes with Dyrk1a**. Transiently transfected HEK-293FT cells. A, E, I) Fluorescence of GFP and fusion proteins. B, F, J) Fluorescence of mRFP1 and fusion proteins. C, G, K) Fluorescence of nuclei stained with DAPI. D, H, L) Composite overlay image of GFP, RFP and DAPI signals. A, B, C, D) GFP and mRFP1 distribute throughout the cytoplasm and nucleus. E, F, G, H) The GFP-Wdr68 fusion and the mRFP1-Dyrk1a fusion are co-localized to the nucleus. I, J, K, L) The GFP-T284F Wdr68 mutant does not co-localize with nuclear mRFP1-Dyrk1a.

The only proteins known to physically interact with Wdr68 are Dyrk1a and Dyrk1b [30]. Dyrk1a has been observed to localize within the nucleus [52]. To determine whether Wdr68 also localizes to the cell nucleus we constructed mRFP1-Dyrk1a and GFP-Wdr68 fusion protein vectors and transiently transfected HEK-293FT cells with various combinations of the vectors [53]. As controls, empty GFP and mRFP1 vectors were both detected throughout the cell (Figure 7A–D). Consistent with previous reports on Dyrk1a localization, the mRFP1-Dyrk1a fusion protein localized to the nucleus when co-expressed with an empty GFP vector (data not shown). The GFP-Wdr68 fusion protein also localized to the nucleus when co-expressed with an empty mRFP1 vector (data not shown). When expressed together, GFP-Wdr68 and mRFP1-Dyrk1a co-localized to the nucleus (Figure 7E–H). In all cases, the locations of nuclei were determined using DAPI.

**Figure 8:**
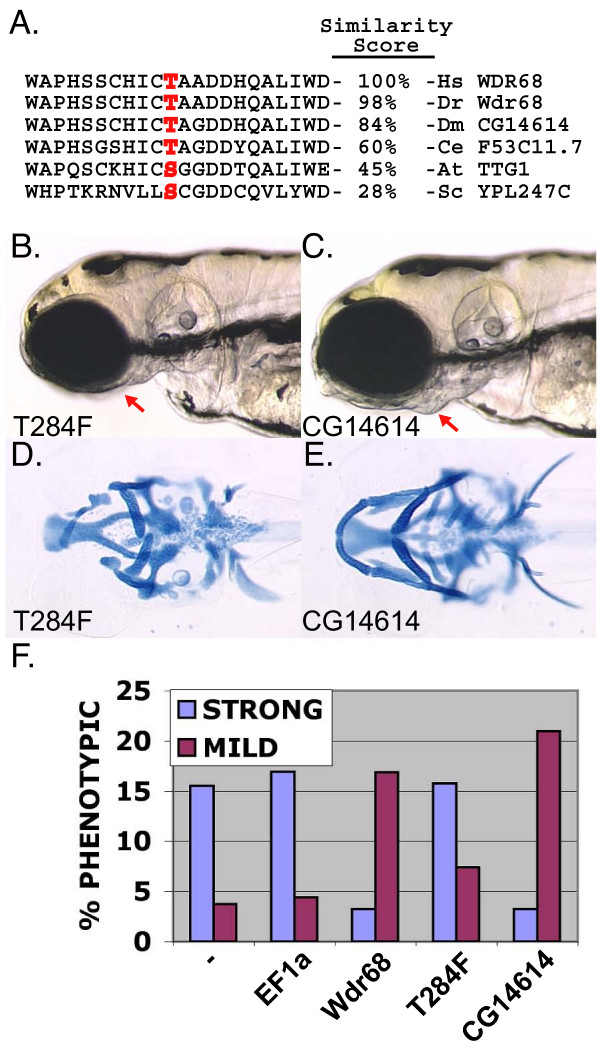
***wdr68 *activity is functionally conserved**. A) Sequence similarity scores generated using ClustalW between *wdr68 *homologs among selected representative organisms. Region shown contains the functionally important position, highlighted in red, identified in the *ttg1-9 *allele. B) The T284F mutant cannot rescue the *dys *mutant phenotype. Live 4dpf animal shown. C) The drosophila homolog *CG14614 *rescues the *dys *mutant phenotype as efficiently as the zebrafish transcripts. D) Alcian blue staining of attempted rescue animals using T284F transcripts. E) Alcian blue staining of animals rescued using the drosophila homolog *CG14614*. F) Chart summarizing the results of a rescue experiment showing that zebrafish *wdr68 *transcripts rescue the strong mutant phenotype, that T284F transcripts fail to rescue, and that *CG14614 *rescues as efficiently as zebrafish *wdr68*.

In Arabidopsis, there are at least 3 *wdr68 *homologs with Clustal W sequence similarity ranging from 54% to 45%. Among these are the protein TTG1, which is essential for root and shoot patterning events including axis patterning of the leaf. Among several strong Arabidopsis *ttg1 *mutant alleles, *ttg1-9 *encodes a point mutation that changes a single serine to a phenylalanine, S282F [28]. This position is within a region of the protein that is highly conserved across all organisms examined and all multicellular animals code for the chemically similar threonine at this position (highlighted in red in Figure 8A). Thus we engineered the corresponding putative null T284F mutation into the zebrafish *wdr68 *gene. To determine whether the engineered putative null T284F can still localize with nuclear Dyrk1a, we constructed a GFP-T284F fusion. The T284F fusion protein failed to co-localize with mRFP1-Dyrk1a (Figure 7I–L).

### *wdr68 *activity is conserved in animals lacking NC cells

BLAST analysis with *wdr68 *revealed the presence of *wdr68 *homologs in all eukaryotic organisms for which complete genome sequence is available including non-vertebrate animals like Drosophila [54]. Using Clustal W to determine sequence similarities [55], the Drosophila homolog, *CG14614*, is 84% similar to the zebrafish gene. To determine whether *CG14614 *is a functional homolog to the vertebrate protein, we developed an RNA rescue assay for the *dys *mutant cartilage phenotype. We injected *dys *mutant clutches with FLAG-tagged zebrafish *wdr68 *transcripts and compared them to either uninjected animals or animals injected with *ef1α *transcripts as a negative control (Table 3 and data not shown). While *ef1α *transcripts were unable to rescue the mutant phenotype, zebrafish *wdr68 *rescued the strong cartilage patterning defects causing a concomitant increase in the number of observed mild joint fusion animals (Figure 8F). We also tested the engineered T284F mutant in the RNA rescue assay. The T284F transcript was unable to rescue the cartilage defects consistent with the prediction that this encodes a null allele similar to the plant homolog (live in Figure 8B, Alcian blue in Figure 8D, graph in Figure 8F). We then tested *CG14614 *for the ability to rescue the cartilage defects present in the *dys *mutant zebrafish. Remarkably, *CG14614 *rescued the strong cartilage defects as efficiently as the zebrafish *wdr68 *transcripts (live in Figure 8C, Alcian blue in Figure 8E, graph in Figure 8F). Thus, *wdr68 *activity is functionally conserved from animals that lack NC cells.

**Table 3:** RNA rescue of the *dys *mutant phenotype.

	Jaw phenotype		
Transcript injected	wild type	strong	mild
none	109 (81%)	21 (16%)	5 (4%)
*ef1α*	107 (79%)	23 (17%)	6 (4%)
*wdr68*	123 (80%)	5 (3%)	26 (17%)
*T284F*	83 (77%)	17 (16%)	8 (7%)
*CG14614*	94 (76%)	4 (3%)	26 (21%)

## Discussion

### Insights from screening for craniofacial mutants

As the zebrafish insertional mutant collection represents approximately 25% of the genes essential during the first 5 days and this screen identified 16 loci, we can extrapolate that at least 80 genes essential during the first 5 days of development are also required for craniofacial development in the zebrafish [6]. Genes essential for very early developmental events such as gastrulation may have been missed in this screen. Similarly, not all defects in craniofacial patterning will necessarily result in lethality by day 5 in the zebrafish. Thus, the range of approximately 80 genes establishes a lower limit on the total number of genes required for craniofacial development.

Previous large-scale ENU screens identified a group of anterior arch mutants with defects in the distal cartilages and most appear to function within the *edn1 *pathway [3,56]. Notably, the *edn1 *pathway controls distal cartilage fate in both the 1^st ^and 2^nd ^arches [10,13]. Neither previous screens nor our screening identified a complementary pathway controlling all proximal cartilage fate. However, 3 of the 6 identified patterning mutants have specific reductions in the proximal 2^nd ^arch derived HM region without affecting the proximal 1^st ^arch PQ implying that distinct pathways control the patterning of the HM region.

Previous studies on the *nos*/*foxI1 *mutant indicated that HM formation requires cell survival signals [14]. Analysis of a zebrafish *integrin-alpha-5 *mutant that displays a reduced aHM region also revealed an essential role for NC cell survival signals [15]. Interestingly, the *wom *and *sec61a *genes might be required for aHM formation due to roles in mediating cell survival through processes typically viewed as general 'housekeeping' activities. At 29% sequence similarity, the closest yeast homolog to *wom *is Swd2p, an essential subunit of the Set1 histone methyltransferase (HMT) complex required for transcription of many genes. Interestingly, differential HMT substrate specificity by different regulatory complexes containing different SET domains is one feature of the proposed 'histone code' for epigenetic mechanisms of gene regulation [57]. Also, Swd2p is a required subunit in the cleavage and polyadenylation factor complex [58-60]. The sec61 complex is essential for protein translocation into the ER. Thus, HM formation may require cell survival signals together with high expression levels of a relatively limited number of apparent housekeeping genes that perhaps mediate rate-limiting steps in survival pathways. Alternatively, paralogs with partial functional redundancy for these presumably cell-essential genes may compensate in other tissues.

### *wdr68 *activity is essential for *edn1*-dependent distal cartilage formation

We showed that *wdr68 *activity is required for *edn1 *expression. We also provided evidence that higher levels of wdr68 activity are required for 1^st ^arch *edn1 *expression and expression of downstream *edn1*-dependent genes such as *hand2 *and *dlx3b *than are required for 2^nd ^arch *edn1 *expression, providing an explanation as to why defects in the 2^nd ^arch appear less severe in *dys *mutants than in *edn1*MO animals. The 2^nd ^arch likely requires lower levels of *wdr68 *activity for induction of the *edn1 *pathway. We also found that *tbx1 *expression was not substantially reduced in *dys *mutants or *wdr68*MO animals suggesting that *wdr68 *either acts downstream of *tbx1 *or in a parallel pathway also required for *edn1 *expression.

### *wdr68 *activity is essential for proximal PQ and PTP formation

Unlike *edn1 *mutants, which retain an identifiable and intact PQ, *dys *mutants display severe disruptions of the PTP and PQ. Thus, *wdr68 *activity is required for all *edn1*-dependent cartilages plus the 1^st ^arch proximal *edn1*-independent PQ region indicating that *wdr68 *activity is required upstream of a putative PQ formation pathway. Future studies will be needed to identify the putative *wdr68*-dependent PQ formation pathway genes. Also, the PTP of the PQ appears to serve as the maxilla region at this early developmental stage. Therefore, it would be very informative to generate and analyze a *wdr68*^-/- ^mouse for defects in the pharyngeal arch derivatives. Such an analysis would also shed substantial light on the evolution of craniofacial structures.

### The Wdr68 and Dyrk1a proteins both localize to the nucleus

The nuclear localization of mammalian Dyrk1a has been previously reported and the biochemical purification of a large protein complex from rabbit skeletal muscle that contains both Wdr68 and Dyrk1a suggests that Wdr68 might also localize to the cell nucleus [30]. Our observation that a zebrafish GFP-Wdr68 fusion indeed localizes to the nucleus is consistent with the formation of an intra-nuclear Wdr68-Dyrk1 protein complex (Figure 7). Since the Wdr68 T284F mutant was not able to complement the craniofacial defects of *dys *mutant animals in the RNA rescue assay, the failure of the Wdr68 T284F mutant to localize to the nucleus may suggest that nuclear localization of Wdr68 is required for craniofacial development. The change from a nuclear to a punctate cytoplasmic localization may also indicate that nuclear localization of Wdr68 requires some additional feature associated with having a hydroxyl group at position 284 such as the ability to accept phosphorylation. However, punctate cytoplasmic expression patterns can also indicate misfolding or destabilization and degradation of a fusion protein. Thus, a more detailed biochemical analysis of the Wdr68-Dyrk1 interaction will be required to determine the true significance of these observed changes.

### The Wdr68-Dyrk1 protein complex is conserved in all eukaryotes

The fly homolog *CG14614 *rescued the strong cartilage defects as efficiently as the zebrafish *wdr68 *transcripts, indicating that the molecular functions of *wdr68 *required by arthopods is the same as that required by vertebrates. Notably, the Wdr68 interaction partners Dyrk1a and Dyrk1b are homologs to the Drosophila *minibrain (mnb) *gene, suggesting conservation of a protein complex [61]. Although the expression pattern for *dyrk1b *has not been reported, in mice, ISH analysis revealed expression of *dyrk1a *in the pharyngeal arches and the *dyrk1a*^-/- ^animals showed severe growth delay and were lethal by E13.5 [31]. Although the *dys *mutants showed only a modest reduction in overall body size, we found that *wdr68 *co-localized in the nucleus with Dyrk1a (Figure 7). Thus, it will be worthwhile to investigate whether the zebrafish *dyrk1 *genes might have roles in craniofacial development. The observation that Dyrk1a intracellular localization undergoes dynamic changes during brain development in the chick suggests that the Wdr68-Dyrk1 complexes may function as signaling intermediates [62]. Likewise, the finding that Dyrk1b is required for the differentiation switch in myotubes suggests that the Wdr68-Dyrk1 protein complexes might function in diverse organisms and tissues to regulate various developmental events [34].

High throughput mass spectrometric identification of protein complexes in yeast identified the Wdr68 homolog YPL247C in 2 complexes, containing 6 and 13 proteins each, and both complexes contain the Dyrk1 homolog YAK1 [63]. Thus, Wdr68-Dyrk1 protein complexes are conserved from yeast to mammals.

Detailed studies of *YAK1 *indicate that it is required for pseudohyphal differentiation and functions in growth control [64,65]. *YAK1 *may act within the Target of Rapamycin (TOR) pathway as rapamycin induces nuclear localization of YAK1 and *YAK1 *deletion confers rapamycin resistance in certain strains [66]. Thus, the presence of *wdr68 *homologs in unicellular eukaryotes such as S. cerevisiae further suggests that Wdr68-Dyrk1 protein complexes participate in switches from growth to differentiation. Further studies will be required to determine how the Wdr68-Dyrk1 protein complexes function and what role they might play in craniofacial development.

## Conclusion

Our data reveal the identity of approximately 25% of the essential genes required for craniofacial development in the zebrafish and demonstrate functional conservation between the fly and fish *wdr68 *homologs. We further show that the *wdr68 *gene is required upstream of the *edn1 *signaling pathway and that it localizes to the cell nucleus. One intriguing possibility is that a nuclear Wdr68-Dyrk1 protein complex may act as a transcriptional co-regulatory complex. Exploring this possibility will require further characterization of the complex as well as downstream putative target genes.

## Methods

### Animal husbandry and Alcian blue cartilage staining

Animals were maintained as described [45]. The Alcian blue staining method used in this study has been previously described [14].

### RT-PCR and whole-mount in situ hybridization analysis

The primers used to compare wild type to mutant animals by RT-PCR and to create plasmids for generating anti-sense probes for ISH are 3812-f1 5'-ATCTTTCAGACCAAATGCGCCGTTG and 3812-r1 5'-CCTCATTCTCCCAGACGAAAGAAG. Total RNA was isolated from equal numbers of either wild type or mutant animals by the TRIZOL method essentially as described by the manufacturer (Invitrogen). First strand cDNA synthesis using a poly dT primer and PCR from this material was performed as previously described using actin primers as controls [14]. The corresponding PCR fragments were subcloned into the pCR-TOPO bluntII (Invitrogen) vector to yield the plasmid pCR-3812. To make the 1^st ^strand cDNA samples for comparison of the various developmental stages, animals were harvested at the appropriate time points and processed by the TRIZOL method. Unfertilized oocytes were harvested by squeezing an anesthetized female and eggs were processed by the TRIZOL method. The primers used to compare *wdr68 *across the various developmental stages are 3812-f2 5'-GATCGCCATTTGCTACAACAACTGC and 3812-r1 5'-CCTCATTCTCCCAGACGAAAGAAG. The primers used for edn1 are edn1-start1 5'-ATGCATTTGAGGATTATTTTCCCAGTTCTGAC-3' and edn1-stop1 5'-CTATGAGTTTTCAGAAATCCACGCTTGG-3'. The primers to compare rpL35 are 258-RTF2 5'-GCTGCTTCCAAGCTCTCAAAAATCC-3' and 258-RTR 5'-TGCCTTGACGGCGAACTTGCGAATG-3'. The ISH method was as previously described with all probes at a final concentration of 200ng/mL [14]. The *edn1 *plasmid has been previously described [13]. The bapx1 plasmid has been previously described [19]. The *hand2 *plasmid has been previously described [67]. The *dlx3b *plasmid has been previously described [68]. The *sox9a *plasmid has been previously described [40].

### Antisense morpholinos used in this study

The morpholino sequences used in this study for the *wdr68 *gene are 3812-1 5'-GAAGCATGTCGTTGCACGGTAAACG-3' (sense control), *wdr68*MO 5'-CGTTTACCGTGCAACGACATGCTTC-3' and *wdr68*MO2 5'-CATGCTTCAATCCCAGTGATCGGCG-3'. The *edn1*-MO sequence has been previously reported [13]. The *moz*-MO3 sequence has been previously reported [51].

Wild type and *dys *mutant clutches were injected with morpholino concentrations ranging from 100 uM to 1 mM for the *wdr68 *sequences or 200 uM for the *edn1*-MO using pulled glass needles and a picospritzer as previously described [14]. The *dys+wdr68*MO animals received an approximate 1 nL injection of 1 mM *wdr68*MO. The *wdr68*MO2 sequence was fully active at 300 uM. Comparable injections with the 3812-1 control sequence had no effect on cartilage formation.

### RNA rescue

The *wdr68 *open reading frame was amplified using primers 3812-FLAGf 5'-taatagaattccaccATGGATTACAAGGATGACGACGATAAGggtATGTCactcCAtGGcAAgCGAAAAGAGATCTACAAATACGAGGCG-3' and 3812-stop1 5'-ctcgagCTACACCCGCAGGATCTCCAGG-3' and subcloned into the pCS2+ vector as an EcoRI-XhoI fragment. The T284F mutation was created by nested amplification of primer T284F-f1 5'-CACCTCATTCCTCCTGCCACATATGTtttGCAGTAGCGGACGATCACCAG -3'with 3812-stop1 and 3812-FLAGf with T284F-r1 5'-CTGGTGATCGTCCGCTACTGCaaaACATATGTGGCAGGAGGAATGAGGTG -3' followed by mixing of the products and further amplification with the outer primers 3812-FLAG-f1 and 3812-stop1. Sequencing confirmed the presence of the T284F mutation subcloned into the pCS2+ vector. The Drosophila homolog *CG14614 *was amplified from 1^st ^strand Drosophila cDNA using primers dmWDR68-f2 5'-ttcttcgaattccaccATGTCCTCGACCGCCGGAAAGC-3' and dmWDR68-r2 5'-ttcttctcgtcgacTTAGACCCGCAGGATCTCGCACG-3', digested with EcoRI and SalI and subcloned into the EcoRI and XhoI sites of the pCS2+ vector. All rescue constructs were linearized using NotI and capped mRNA was synthesized using the mMessage mMachine kit essentially as described by the manufacturer (Ambion). The xenopus *ef1a *control transcript was supplied as the control template in the kit. All mRNA were quantified and ran on an agarose gel to confirm synthesis. Mutant clutches were injected as described for the morpholinos with approximately 1 nL of a 5 ng/uL solution of each RNA containing 0.1% phenol red. Animals were processed for Alcian blue staining at 4dpf.

### Intracellular localization

The GFP open reading frame from pEGFP-C2 (Clontech) was subcloned as a Eco47III-EcoRI fragment into the ClaI(blunted)-EcoRI sites of the pCS2+WDR68 vectors to yield pCS2+GFP-WDR68 and pCS2+GFP-T284F. The mRFP1 open reading frame was PCR amplified using primers mRFP1-start 5'-ttcttgaattccaccATGGCCTCCTCCGAGGACG-3' and mRFP1-nonstop 5'-ttcttcttctcgagagatctgatatcGGCGCCGGTGGAGTGGCGGCCCT-3' from a bacterial expression vector [53]. The mRFP1 PCR fragment was subcloned into pCS2+ as an EcoRI-XhoI fragment. The mouse DYRK1A coding sequence was amplified from E17 stage mouse embryo total RNA using primers mh-DYRK1A-f1 5'-ttcttcttagatctATGCATACAGGAGGAGAGACTTCAGC -3' and mh-DYRK1A-r1 5'-ttcttcttctcgagTCACGAGCTAGCTACAGGACTCTGTTG-3'. The DYRK1A PCR fragment was subcloned into pCS2+mRFP1 as a BglII-XhoI fragment.

Transient transfection of the fluorescent protein fusion plasmids into HEK-293FT cells grown in glass chamber slides was performed using Lipofectamine 2000 (Invitrogen) as per the manufacturers protocol. Cells were fixed and visualized approximately 20–24 hours post-transfection.

## Authors' contributions

RN designed the study, carried out the experiments, analyzed the data and drafted the manuscript. AA cloned the mutated loci, participated in and coordinated the initial screen from which the screened mutants were isolated, participated in the coordination of the study and in drafting the manuscript. NH conceived of the study and helped draft the manuscript.

## Supplementary Material

Additional file 1 **The chondrocyte differentiation and cell morphology class mutants**. A, B, E, F, I, J, M, N) Head morphology at 4dpf. C, D, G, H, K, L, O, P) Alcian blue stained pharyngeal cartilages at 4dpf viewed ventrally, except for panel (P) which is flat mounted for comparison with figure 1. A, C) Wildtype. B, D) *b3gat3*^hi307 ^mutant. E, G) *uxs1*^hi954 ^mutant. F, H) *slc35d1*^hi3378 ^mutant. I, K) *atp6ap2*^hi3681 ^mutant. J, L) *b4galt7*^hi4063A ^Ehlers-Danlos mutant. M, O) *srp68*^hi4153 ^mutant. N, P) *odk*^hi1042 ^cell morphology mutant. The *jef/sox9a*, *goz/mbtps1*, *kny/glp6 *mutants have been previously described and so are omitted here. Click here for file
