# Supplementary figures and images for: A zebrafish screen for craniofacial mutants identifies wdr68 as a highly conserved gene required for endothelin-1 expression
Source: BMC Dev Biol. 2006 Jun 7;6:28. doi: 10.1186/1471-213X-6-28 (PMC1523201; doi:10.1186/1471-213X-6-28)

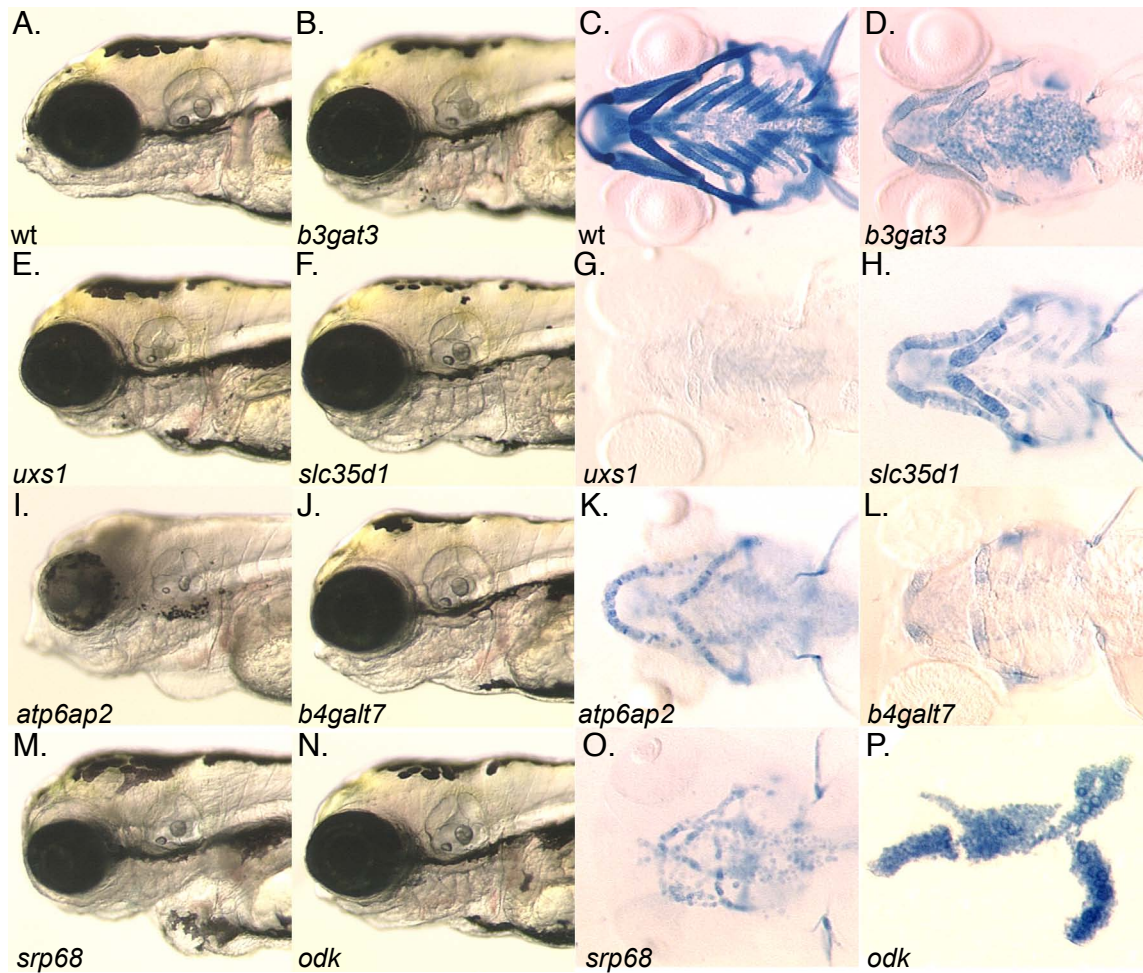

Supplement: Additional file 1 — The chondrocyte differentiation and cell morphology class mutants. A, B, E, F, I, J, M, N) Head morphology at 4dpf. C, D, G, H, K, L, O, P) Alcian blue stained pharyngeal cartilages at 4dpf viewed ventrally, except for panel (P) which is flat mounted for comparison with figure 1. A, C) Wildtype. B, D) b3gat3hi307 mutant. E, G) uxs1hi954 mutant. F, H) slc35d1hi3378 mutant. I, K) atp6ap2hi3681 mutant. J, L) b4galt7hi4063A Ehlers-Danlos mutant. M, O) srp68hi4153 mutant. N, P) odkhi1042 cell morphology mutant. The jef/sox9a, goz/mbtps1, kny/glp6 mutants have been previously described and so are omitted here. [file 1471-213X-6-28-S1.pdf]
